# Childhood maltreatment and joint trajectories of social isolation and depression as predictors of cognitive impairment in late midlife: a prospective cohort study

**DOI:** 10.1017/S204579602610081X

**Published:** 2026-07-22

**Authors:** Diego A. Díaz-Faes, Molly Maxfield, Cathy S. Widom

**Affiliations:** 1 Department of Epidemiology, Columbia University, Mailman School of Public Health, New York, NY, USA; 2 Center for Innovation in Healthy and Resilient Aging, Edson College of Nursing and Health Innovation, Arizona State University, Phoenix, AZ, USA; 3 Psychology Department, John Jay College, City University of New York, New York, NY, USA; 4 Graduate Center, City University of New York, New York, NY, USA

**Keywords:** child abuse and neglect, cognitive impairment, cohort study, depression, life course epidemiology, social isolation

## Abstract

**Aims:**

Childhood maltreatment has been associated with increased risk of cognitive impairment in later life, but little is known about whether distinct joint trajectories of social isolation and depression across midlife predict this risk.

**Methods:**

Using a prospective cohort design with documented childhood maltreatment and matched controls (*N* = 1196) followed from ages 0 to 11 into late midlife, we modelled joint trajectories of social isolation and depression (ages 29–47) using group-based trajectory modelling (GBTM) and examined their associations with cognitive impairment with no dementia (CIND) at age 59 through modified Poisson regression, controlling for risk factors related to neurodegenerative outcomes (i.e., hypertension, diabetes, obesity, limited physical activity, hearing impairment, smoking, excessive alcohol consumption and traumatic brain injury).

**Results:**

GBTM identified four groups: ‘Not isolated or depressed’ (67.7%), ‘Moderately isolated’ (11.7%), ‘Moderately depressed’ (16.5%) and ‘Chronically isolated and depressed’ (4.1%). Using the ‘Not isolated or depressed’ group as the reference category, membership in the highest-risk trajectory (‘Chronically isolated and depressed’) was associated with a 41.5% higher risk of CIND, whereas the ‘Moderately isolated’ and ‘Moderately depressed’ groups did not differ significantly from the reference group. Black non-Hispanic participants and those with documented childhood maltreatment histories had 32.8% and 21.2% higher risk of CIND, respectively, and each additional year of education was associated with a 4.9% reduction in CIND risk.

**Conclusions:**

Sustained co-occurring social isolation and depression from early to mid-adulthood predicted increased risk of CIND independent of established risk factors, highlighting prolonged social and emotional difficulties as modifiable targets for late midlife cognitive health.

## Introduction

Mild cognitive impairment (MCI) is a frequently used term to describe the stage between normal cognitive function and dementia (Roberts and Knopman, 2013). MCI is estimated to affect between 15% and 20% of individuals aged 60 and older worldwide (Petersen, 2016). Other estimates suggest that approximately two-thirds of the US population experience some level of MCI at an average age of about 70 years (Hale *et al.*, 2020). Prospective longitudinal studies have shown that the early experience of child maltreatment can disrupt cognitive functioning, increasing the risk of MCI and dementia in later life (Geoffroy *et al.*, 2016; Danese *et al.*, 2017; Widom *et al.*, 2023; Danese and Widom, 2024; Assuras *et al.*, 2025). The cognitive consequences of childhood maltreatment may manifest decades later and follow many different developmental pathways, altering interconnected and interacting systems and causing a spillover effect. The spillover effect may occur directly in a unidirectional or bidirectional manner, or indirectly through third variables that, in turn, shape later outcomes (Masten and Cicchetti, 2010).

Childhood maltreatment may accelerate cognitive decline by triggering biological responses that impact brain development and increase the risk of stress-related diseases in adulthood. These short- and long-term mechanisms include toxic stress and dysregulated stress responses (Shonkoff *et al.*, 2012), prolonged activation of allostatic systems leading to detrimental physiological effects (Danese and McEwen, 2012) and early functional adaptations to child adversity that increase latent vulnerability to future stressors (McCrory *et al.*, 2017). These responses may disrupt brain development trajectories, especially during sensitive periods, affecting sensory systems and neural circuits involved in threat detection, emotional regulation and reward processing (Teicher *et al.*, 2016). As a result, these stress responses can alter brain structure, function, connectivity and network architecture, potentially impairing cognitive functioning (Twardosz and Lutzker, 2010; Teicher *et al.*, 2016; Li *et al.*, 2023).

Beyond its biological and neurodevelopmental impact, longitudinal evidence has shown that child maltreatment heightens cognitive, emotional, social and behavioural risks (Gilbert *et al.*, 2009), increasing the likelihood of difficulties in establishing and maintaining social relationships (de Heer *et al.*, 2024), depression (Gardner *et al.*, 2019) and cognitive impairment later in life (Su *et al.*, 2019). Longitudinal evidence has shown that social isolation accelerates cognitive decline and is associated with incident MCI and dementia (Kuiper *et al.*, 2016; Evans *et al.*, 2019; Piolatto *et al.*, 2022; Laustsen *et al.*, 2024). Similarly, depression is also linked to deficits in cognitive function, MCI and dementia (O’Brien, 2004; Rock *et al.*, 2014; Veronese *et al.*, 2024). Together, social isolation and depression can reinforce each other over time (Yu *et al.*, 2015; Do and Widom, 2024), potentially creating a self-perpetuating cycle that further elevates the risk of cognitive decline.

At the sociodemographic level, several factors are central to understanding heterogeneity in MCI. Sex differences in MCI are unclear and not fully characterized across different stages of cognitive decline. However, men and women in the US appear to follow different risk pathways for developing MCI and progressing to dementia, with women tending to have higher baseline cognitive performance than men but faster rates of decline in global cognition and executive function (Levine *et al.*, 2021). Racial and ethnic disparities in MCI and dementia are well documented. Black and Hispanic individuals have consistently higher rates of MCI and dementia than White individuals (Mayeda *et al.*, 2016; Wright *et al.*, 2021). Beyond prevalence differences, Black and Hispanic individuals tend to experience an earlier age of cognitive impairment onset and accumulate more years living with impairment (Hale *et al.*, 2020). Education is an established protective factor for cognitive decline (Gauthier *et al.*, 2006; Livingston *et al.*, 2024). Consistent with cognitive reserve theory (Stern, 2002), longitudinal studies have shown that higher education reduces the risk of later cognitive impairment (Zhu *et al.*, 2017; Clouston *et al.*, 2020).

Theory and prior evidence point to qualitatively distinct courses of depression and social isolation over time, rather than a single homogeneous path. Prior longitudinal work on each construct separately suggests that individuals tend to follow a small number of prototypical trajectories, typically between three and four groups (Musliner *et al.*, 2016; Xiang *et al.*, 2021), which may differentially shape cognitive ageing. Some longitudinal studies have examined the impact of social isolation or depression trajectories on later cognitive function (Formánek *et al.*, 2020; Lay-Yee *et al.*, 2023). However, no known studies have addressed the effects of joint trajectories of social isolation and depression on later cognitive functioning using an objective measure of child maltreatment and a control group, with proper assessment of the temporal order of events and an extended follow-up period.

This study address this gap by leveraging data from a prospective cohort designed to assess the consequences of child maltreatment. We examined whether childhood maltreatment and joint trajectories of social isolation and depression from young adulthood through midlife predicted cognitive impairment with no dementia (CIND) at age 59. We hypothesized that: (H1) individuals with a documented history of childhood maltreatment would have a greater risk of CIND at age 59 than those without such a history; and (H2) membership in the highest-risk social isolation and depression trajectory between ages 29 and 47 would be associated with a greater risk of CIND at age 59 than membership in the no-risk trajectory.

## Method

### Design and participants

This study draws on data from a prospective investigation that tracked a cohort of abused and neglected children, along with demographically matched controls, into adulthood (Widom, 1989a, 1989b). Cases of child abuse and neglect were identified through records from county juvenile and adult criminal courts in a Midwestern metropolitan area between 1967 and 1971. To ensure a clear understanding of the exposure, causal relationship and temporal sequence, only cases where the children were under 12 years at the time of the incidents were considered. The original archival study included 908 abused and neglected children, who were matched with non-abused, non-neglected children. The matching process was based on age, sex, race/ethnicity and approximate family social class during the study period. Children with any official record of abuse or neglect were excluded as controls. Matches were found for 73% of the abused and neglected children, resulting in 667 controls. The data utilized in this paper comes from Interviews 1 to 5. Table 1 presents descriptive statistics for the participants and the main study variables at the different time points. Despite attrition, there were no significant differences in terms of age, race or maltreatment status across the study waves. However, females were more likely to remain in the study at Interviews 2 (*p* = .004), 3 (*p* < .001), 4 (*p* < .001) and 5 (*p* < .001).

**Table 1.** Descriptive characteristics of participants across the study waves Table 1 long description.

	Records		Interviews			
	1967–1971	1 (1989–1995)	2 (2000–2002)	3 (2003–2005)	4 (2009–2010)	5 (2022–2023)
	*N* = 1575	*N* = 1196	*N* = 896	*N* = 807	*N* = 649	*N* = 447
	*M* (*SD*)	*M* (*SD*)	*M* (*SD*)	*M* (*SD*)	*M* (*SD*)	*M* (*SD*)
Mean age	6.4 (3.3)	29.2 (3.8)	39.5 (3.5)	41.2 (3.5)	47.0 (3.5)	59.4 (3.6)
Mean age at petition	6.4 (3.3)	6.3 (3.3)	6.2 (3.3)	6.3 (3.3)	6.4 (3.3)	6.0 (3.4)
Education	–	11.5 (2.2)	12.2 (7.2)	12.2 (7.5)	12.0 (5.1)	11.7 (2.3)
Social isolation	–	2.6 (1.2)	1.3 (1.4)	2.2 (1.3)	1.7 (1.1)	1.6 (1.5)
Depression	–	3.4 (2.7)	12.8 (11.1)	11.4 (11.4)	13.2 (11.6)	16.3 (8.4)
	%	%	%	%	%	%
Mild cognitive impairment	–	–	–	–	–	56.6
Sex, female	50.7	48.7	51.0	52.7	53.9	56.7
White non-Hispanic	66.2	62.9	62.2	60.4	59.2	58.8
Black non-Hispanic	32.6	34.9	35.2	37.3	34.5	35.6
Hispanic	0.3	3.8	4.0	4.0	3.7	3.1
Other race-ethnicity	1.1	2.3	2.2	2.6	2.6	2.5
Childhood maltreatment	57.7	56.5	55.8	56.8	55.2	56.8
Physical abuse	10.2	9.2	8.8	9.7	8.3	9.2
Sexual abuse	9.7	8.0	7.6	7.5	8.3	8.3
Neglect	44.3	45.4	45.3	45.9	44.1	45.4

*Note:* Mean age at petition = mean age at which childhood maltreatment was identified in court records. Depression symptoms at Interview 1 were assessed using the National Institute of Mental Health Diagnostic Interview Schedule – Revised (DIS-III-R) (Robins et al., 1989), which permits Diagnostic and Statistical Manual of Mental Disorders -III-R (DSM-III-R) diagnoses (American Psychiatric Association, 1987). For subsequent interviews, the Center for Epidemiologic Studies Depression Scale (CES-D) was used to assess depression symptoms (Radloff, 1977).

### Procedures

Participants were interviewed in person at a location of their choosing. Both the interviewers and the participants were kept unaware of the study’s specific goals, the participants’ group classifications and the presence of a group with a history of child abuse and/or neglect. Participants were told they were selected to be part of a study focusing on a large group of people who grew up in the same area during the late 1960s and early 1970s. Institutional Review Board approval was obtained at each wave of the study, and participants were provided with written informed consent. This study was approved by the Human Research Protection Program at The City University of New York (Protocol #: 2015-0133). For individuals with limited reading ability, the consent form was presented and explained verbally.

## Measures and variables

### Childhood maltreatment

Childhood maltreatment was determined by reviewing official family and adult criminal court records from 1967 to 1971, covering the period when the children were between the ages of 0 and 11 years. Physical abuse cases involved injuries such as bruises, burns, lacerations, fractures and other physical harm. Sexual abuse cases included serious offences like felony sexual assault, rape, sodomy, fondling and incest. Neglect cases represented extreme failure by parents to meet caregiving standards deemed acceptable by the community and professionals at the time, and included failure to provide adequate food, clothing, shelter and medical attention. Childhood maltreatment was operationalized as a binary variable based on whether individuals had experienced any form of documented abuse or neglect.

### Depression

Depression was assessed in Interviews 1–4, at ages 29, 39, 41 and 47. Depression symptoms at Interview 1 were evaluated using the National Institute of Mental Health (NIMH) Diagnostic Interview Schedule-III – Revised (DIS-III-R) (Robins *et al.*, 1989), permitting diagnoses based on *Diagnostic and Statistical Manual of Mental Disorders-III – R* (DSM-III-R) (American Psychiatric Association, 1987). The DIS-III-R, a highly structured interview schedule intended for use by non-professional interviewers, has shown good reliability (Helzer, 1985). We utilized lifetime symptom scores to measure the severity of depression symptoms, with higher scores indicating a greater number of symptoms. Computer programs designed for scoring the DIS-III-R were employed to calculate the total number of depression symptoms, ranging from 0 to 9 (*M* = 3.4, *SD* = 2.7; α = .87). In Interviews 2–4, depression was assessed through the *Center for Epidemiologic Studies Depression Scale* (CES-D) (Radloff, 1977). The CES-D is a 20-item self-report measure with high internal consistency applicable to both general and psychiatric populations. Participants were asked to rate how they felt over the past week on a 4-point Likert scale, ranging from rarely or none of the time (less than 1 day) to most or all of the time (5–7 days). Total scores in CES-D could range from 0 to 60. Higher scores indicate a greater presence of depressive symptoms (α = .90). Due to the variation in how depression symptoms were measured between the initial interview and later time points, depression scores for each wave were standardized to *z*-scores (Pacheco *et al.*, 2023), with a mean of 0 and a standard deviation of 1.

### Social isolation

Social isolation was also assessed in Interviews 1–4, at mean ages of 29, 39, 41 and 47, respectively. Participants were asked seven questions about the extent of their participation in social activities, including how often they got together with family members, other people for a hobby or leisure activities, close friends, and neighbours, or to attend a church/synagogue/prayer group (Kulka *et al.*, 1990). Each question offered an eight-point Likert-scale response: daily, several times per week, once per week, several times per month, once per month, several times per year, once a year or less and never. Responses were coded as 1 if the participant indicated that they never engaged in that activity, with all other responses coded as 0. Additionally, respondents were asked whether they were married/living with someone (coded as 0) or not (coded as 1). The sum of these seven items represented the level of social isolation, with higher scores indicating greater social isolation (*M* = 2.16, *SD* = 1.10, range = 0–7, α = .66). The scores were standardized to facilitate joint interpretation with depression (*M* = 0; *SD* = 1).

### Cognitive impairment

Recent work has noted that MCI lacks a single, consistently applied diagnostic criterion and conventional diagnostic criteria may be susceptible to false-positive diagnoses and false-negative errors (Kim *et al.*, 2024). We defined CIND using a research-based classification that integrated standardized neuropsychological test norms with functional status. Specifically, CIND was defined as cognitive impairment on standardized neuropsychological tests in the absence of functional impairment (Petersen *et al.*, 1999; Chertkow *et al.*, 2007), rather than solely on the basis of a global clinical rating.

Cognitive functioning was evaluated at a mean age of 59 (Interview 5) using a comprehensive neuropsychological assessment battery consisting of 12 tests: *Benson Complex Figure Copy; Consortium to Establish a Registry for Alzheimer’s Disease (CERAD) neuropsychological battery tests* (Morris *et al.*, 1989): *CERAD Category Fluency Test: Animals, CERAD Word List Learning and Recall (Immediate)* and *CERAD Word List Recall – Delayed.* Verbal Fluency: *Phonemic Letter Fluency (S); Montreal Cognitive Assessment* (Nasreddine *et al.*, 2005); *Multilingual Naming Test* (Gollan *et al.*, 2012); *Number Span Test; Symbol-Digit Modalities Test* (Smith, 1973); *Trail Making Test (A and B*) (Strauss *et al.*, 2006); *WAIS-III Matrix Reasoning* (Wechsler, 1997); *Wide Range Achievement Test, Third Edition (WRAT3)*; and *Stroop Color and Word Test* (Golden *et al.*, 2002). These tests were selected based on previous work, using population-based norms to determine CIND (Smith *et al.*, 1992; Lucas *et al.*, 2005; Steinberg *et al.*, 2005; Sheridan *et al.*, 2006).

Participants also completed the *Functional Activities Questionnaire* (FAQ) (Pfeffer *et al.*, 1982), which involved 10 common daily living activities, such as shopping independently, keeping appointments and travelling outside the neighbourhood. They were asked whether, in the past 4 weeks, they had encountered any difficulty or required assistance with each task. Response categories included: normal, difficulty but able to perform alone, need assistance or fully dependent (coded from 0 to 3).

Cognitive status was determined following the approach described in our previous work (Assuras *et al.*, 2025). That work also reports and compares test scores across groups. Raw cognitive test scores were transformed into *T* scores using population-based norms (Mayo’s Older Americans Normative Studies and Mayo’s Older African-Americans Normative Studies) (Smith *et al.*, 1992; Lucas *et al.*, 2005; Steinberg *et al.*, 2005; Sheridan *et al.*, 2006), Offspring study (Manly *et al.*, 2020) and test manuals (Wechsler, 1997); raw scores were retained when norms were unavailable. As described above, functional dependence was assessed with the FAQ. Participants were categorized as CIND if a normative test score was ≥1.5 SD below the population mean (*T* < 35) and they did not meet the FAQ cutpoint for functional dependence (FAQ < 9). Participants who met the criteria for dementia (*n* = 10), defined as meeting the CIND criterion together with functional dependence (FAQ ≥ 9, i.e., dependent in ≥3 activities), were excluded.

### Covariates

Following established literature, hypertension, diabetes, obesity, limited physical activity, hearing impairment, smoking, excessive alcohol consumption and traumatic brain injury were included as covariates given their documented associations with cognitive and neurodegenerative outcomes (Livingston *et al.*, 2024), captured at Interview 3 (*M* age 47) (Widom *et al.*, 2023). We also controlled for sex, age, race/ethnicity and years of education. Sex was coded as a binary variable, male = 0 and female = 1. Age refers to the age in years at the time of Interview 1 (*M* = 29.2, *SD* = 3.8). Race/ethnicity included individuals who self-identified as White non-Hispanic (*n* = 735, 61.5%), Black non-Hispanic (*n* = 389, 32.5%), Black-Hispanic (*n* = 28, 2.3%), White Hispanic (*n* = 17, 1.4%), American Indian (*n* = 21, 1.8%), Pacific Islander (*n* = 1, 0.1%) and Others (*n* = 5, 0.4%). Due to the lower prevalence, they were combined as White non-Hispanic, Black non-Hispanic, Hispanic (*n* = 45, 3.8%) and Other (*n* = 27, 2.3%). Education refers to the number of years of school completed, measured at the time of Interview 1 (*M* = 11.5; *SD* = 2.2).

## Statistical analysis

To find the latent trajectories of social isolation and depression, we employed group-based trajectory modelling (GBTM), particularly a joint trajectory model (Nagin *et al.*, 2018). GBTM is a special case of finite mixture modelling that aims to uncover clusters of individuals exhibiting similar patterns in the progression of specific measures over time (Nagin *et al.*, 2024). This semi-parametric method is often useful in clinical research or when more complex models, such as growth mixture models that capture within-group variability, are not feasible (van der Nest *et al.*, 2020; Nagin *et al.*, 2024). The models were estimated using robust maximum likelihood with 2000 random starts and 500 iterations. We included the quadratic term to capture nonlinear trajectories.

Then, we estimated GBTM models with two to seven latent groups and selected the optimal number of trajectory groups based on the following fit indices and classification indicators (Nylund *et al.*, 2007; van de Schoot *et al.*, 2017). Lower values of the Akaike information criterion (AIC), Bayesian information criterion (BIC) and sample size adjusted BIC (SSABIC) indicate a better fit. The adjusted Lo-Mendell-Rubin likelihood ratio test (Adj. LMR-LRT) and bootstrapped likelihood ratio test (BLRT) compare two adjacent models, with a significant *p*-value (*p* < .05) reflecting that the *k*-class model fits better than the *k* − 1 class model. We also examined classification accuracy using entropy and average posterior probabilities (AvePPs), minimum group size (at or over 5%) and theoretical interpretability.

Next, we summarized the selected solution and compared the four GBTM groups on key characteristics. For continuous variables (age at Interview 5 and years of education), we tested overall group differences with pooled *F* tests and reported eta-squared (η^2^) as the effect size. For categorical variables (sex, race/ethnicity, childhood maltreatment and CIND), we calculated χ^2^ statistics, reporting Cramér’s *V* for omnibus tests and Phi (φ) for pairwise comparisons. Tests were pooled across imputations using the D_1_ procedure for continuous variables and the D_2_ procedure for categorical variables (Van Buuren, 2018). Effect sizes were computed within each imputed dataset and averaged across imputations. Pairwise comparisons were adjusted using the Holm–Bonferroni method.

Finally, we regressed CIND at age 59 on childhood maltreatment and joint social isolation and depression trajectory-group membership, adjusting for the mentioned covariates. The trajectory groups identified in the GBTM were entered as dummy variables, with the ‘Not isolated or depressed’ group (Group 1) as the reference category. Because the prevalence of CIND in this cohort was high (57.3%), the rare-disease assumption (event < 10%) did not hold; we employed the modified Poisson regression to avoid overestimation (Davies *et al.*, 1998; Zou, 2004), and applied Rubin’s rules to pool parameter estimates (Rubin, 2004). Additionally, to contrast the predictive power of individual snapshots of social isolation and depression, we also conducted four modified Poisson regressions using social isolation and depression in each interview as predictors of CIND at age 59. We restricted the social isolation and depression measures to the first four interviews so that all predictors temporally preceded the CIND outcome at Interview 5.

We visually inspected the missing data pattern and assumed a missing at random (MAR) distribution. All statistical tests were two-sided at a significance level of 5%. Analyses were conducted in R version 4.5.1 (R Core Team, 2025). We used the mice package for multiple imputations (van Buuren and Groothuis-Oudshoorn, 2011) and the MplusAutomation package for the GBTM (Hallquist and Wiley, 2018). As sensitivity analyses to assess possible departures from the MAR assumption, we conducted a delta-adjustment analysis within the multiple-imputation framework (Leacy *et al.*, 2017), adding δ to the linear predictor of the logistic imputation model for CIND for participants with missing CIND at Interview 5. We set exp(δ), the odds ratio of CIND for missing versus observed outcomes, to 1.5, 2.0 and 3.0, and refitted the modified Poisson model at each δ.

## Results

### Models’ solution and selection

Table 2 shows all the model adequacy criteria we relied on to select the best-fitting solution. Based on all criteria, parsimony and interpretability, the 4-group solution was selected. The 4-group solution identified conceptually distinct and theoretically meaningful trajectories across the groups and demonstrated the best performance among all solutions. It shows a significant model improvement over the 3-group model (ALMR-LRT *p* < .0073 and BLRT *p* < .001), indicating a better fit and lower values in the information criteria (AIC, BIC and SSABIC). It also retained an acceptable entropy (.721), while the smallest group comprised about 4% of the sample and is theoretically relevant. In contrast, the 5-group solution, despite lower information criteria values, as is common when increasing the number of latent groups and a statistically significant LMR-LRT, suggested an overfitted solution due to the presence of two very small groups and did not identify an additional theoretically meaningful group. The 6- and 7-group solutions did not outperform the preceding models (Adj. LMR-LRT *p* > .05), making the 4-group model the optimal balance between fit and interpretability.

**Table 2.** Model-fit model adequacy criteria for GBTM solution Table 2 long description.

Solution	AIC	BIC	SSABIC	Adj. LMR-LRT *p*	BLRT *p*	Entropy	Smallest group %	AvePPs
Two-groups	19,091.364	19,198.185	19,131.481	<.001	<.001	.781	16.30	.903–.934
Three-groups	18,898.978	18,898.978	18,952.468	.0885	<.001	.768	4.10	.834–.911
**Four-groups**	**18,733.195**	**18,911.230**	**18,800.057**	**.0073**	**<.001**	**.721**	**4.10**	**.764–.904**
Five-groups	18,647.385	18,861.028	18,727.620	.0097	<.001	.725	3.51	.749–.917
Six-groups	18,553.124	18,802.374	18,802.374	.2966	<.001	.752	3.70	.766–.899
Seven-groups	18,488.978	18,488.978	18,773.835	.1392	<.001	.748	1.92	.739–.916

*Note: The best solution appears in bold.* LL = log-likelihood, AIC = Akaike’s information criterion, BIC = Bayesian information criterion, SSABIC = sample size adjusted BIC, Adj. LMR-LRT *p* = adjusted Lo-Mendell-Rubin likelihood ratio test, BLRT = bootstrap likelihood-ratio test, AvePPs = average posterior probabilities.

### Description and comparison of trajectory groups

**Figure 1.**
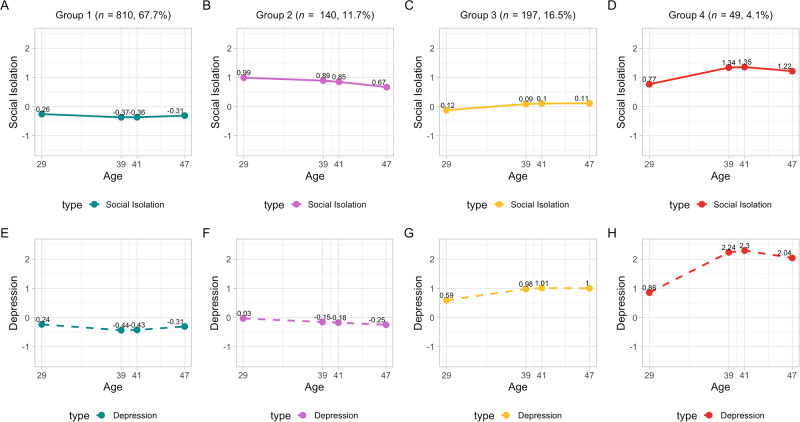
Trajectories of social isolation and depression from early to mid-adulthood. Figure 1 long description.

*Note*. A, Group 1: ‘Not isolated or depressed’; B, Group 2: ‘Moderately isolated’; C, Group 3: ‘Moderately depressed’; and D, Group 4: ‘Chronically isolated and depressed’

The 4-group solution reveals distinct trajectories for social isolation and depression across the GBTMs (Figure 1). Group 1, ‘Not isolated or depressed’, is the no-risk trajectory for social isolation and depression and comprises the majority of the sample (*n* = 810, 67.7%) and was used as the reference group for comparisons. Both social isolation and depression remained slightly below the sample mean, with slight variation across all four ages.

Group 2, ‘Moderately isolated’, represents a low- to mid-risk trajectory for social isolation and depression, encompassing 11.7% of the sample (*n* = 140). The baseline level of social isolation at age 29 is the highest among the groups, but decreases over time, especially between ages 41 and 47. Depression starts around the mean at age 29 and consistently decreases throughout life until reaching the lowest level above average at age 47. Both trajectories follow a mild negative linear pattern.

Group 3, ‘Moderately depressed’, accounts for another low-to-intermediate-risk trajectory and comprises 16.5% of the sample (*n* = 197). This trajectory moves in the opposite direction to Group 2, with a minimal but steady level of social isolation, and with depression moderately increasing until age 41 and then stabilizing at age 47.

Group 4, ‘Chronically isolated and depressed’, is the highest-risk trajectory for social isolation and depression but also the smallest group, including 4.1% of the sample (*n* = 49). Both social isolation and depression exhibit similar curvilinear trajectories, with the highest levels of both variables across all interviews. There is a high increase in social isolation and depression from age 29 to 39, followed by a slight increase in the intermediate waves at ages 39 and 41, and a noticeable decrease at age 47. However, the values and steepness of the trajectory are significantly greater and more pronounced for depression compared to social isolation.

After assigning participants to their most likely groups, we compared the characteristics of the four groups across variables of interest (Table 3). Among the continuous variables, one-way ANOVA showed significant differences only in years of education (D_1_ = 20.09, *p* < .001, η^2^ = .049). Pairwise comparisons indicated that Group 1 had more years of education than Group 2 (*p* < .001), Group 3 (*p* = .011) and Group 4 (*p* < .001), and that Group 3 had more years of education than Group 2 (*p* = .011) and Group 4 (*p* < .001). Descriptively, Group 1 had the highest years of education, and Group 4 had the lowest. No significant overall differences were observed in age. Omnibus tests revealed significant differences across the four GBTM groups in sex (D_2_ = 8.63, *p* < .001, φc = .147) and childhood maltreatment history (D_2_ = 13.89, *p* < .001, φc = .187), but not in race/ethnicity. Corrected pairwise comparisons for sex showed that Group 3 differed significantly from Group 1 (*p* < .001, φ = .150) and Group 2 (*p* = .002, φ = .195). For childhood maltreatment, Group 1 differed significantly from Group 2 (*p* < .001, φ = .166) and Group 3 (*p* < .001, φ = .141) after correction.

**Table 3.** Participants’ characteristics by joint social isolation and depression trajectory group Table 3 long description.

	Group 1	Group 2	Group 3	Group 4
	*n* = 810 (67.7 %)	*n* = 140 (11.7 %)	*n* = 197 (16.5 %)	*n* = 49 (4.1 %)
Age at Interview 5 *M* (*SD*)	59.8 (0.1)	59.5 (0.3)	60.0 (0.2)	60.3 (0.5)
Sex, female (%)	45.1	44.3	64.0	59.2
White non-Hispanic (%)	62.2	55.0	62.4	63.3
Black non-Hispanic (%)	31.4	39.3	32.5	32.7
Hispanic (%)	4.6	2.9	2.0	0.0
Other race-ethnicity (%)	1.85	2.86	3.05	4.08
Education *M* (*SD*)	11.8 (0.1)	10.7 (0.2)	11.3 (0.1)	10.1 (0.4)
Childhood maltreatment (%)	50.1	73.2	67.2	64.6
Physical abuse (%)	7.9	17.4	8.9	10.4
Sexual abuse (%)	6.8	9.4	10.4	16.7
Neglect (%)	40.2	61.6	57.8	45.8
Age at petition *M* (*SD*)	6.3 (3.3)	6.1 (3.3)	6.5 (3.3)	6.5 (3.0)
Hypertension (%)	44.4	44.2	49.0	44.1
Diabetes (%)	15.5	17.8	27.1	22.6
Obesity (%)	41.2	39.1	38.5	48.7
Limited physical activity (%)	10.2	16.5	34.0	51.3
Hearing impairment (%)	11.3	14.5	16.9	31.3
Smoking (%)	51.6	51.6	58.8	59.0
Excessive alcohol consumption (%)	5.2	4.4	7.2	7.9
Traumatic brain injury (%)	3.2	8.8	7.2	23.1
CIND (%)	55.4	66.3	52.8	81.8

*Note:* Age at petition = age at which child maltreatment was identified in the official records; CIND = cognitive impairment with no dementia.

Among the cognitive and neurodegenerative risk factors, significant overall differences were observed for physical limitation (D_2_ = 23.63, *p* < .001, φc = .295) and traumatic brain injury (D_2_ = 5.24, *p* = .001, φc = .166). No significant overall differences were observed for hypertension, diabetes, obesity, hearing impairment, smoking or excessive alcohol use. After correction, pairwise comparisons for physical limitation were significant between Groups 1 and 3 (*p* < .001, φ = .256), Groups 1 and 4 (*p* < .001, φ = .275), Groups 2 and 3 (*p* = .004, φ = .201) and Groups 2 and 4 (*p* < .001, φ = .355). For traumatic brain injury, only Groups 1 and 4 differed significantly after correction (*p* < .001, φ = .190). Finally, CIND differed between groups (D_2_ = 4.34, *p* = .005, φc = .143). Corrected pairwise comparisons showed significant differences between Groups 1 and 4 (*p* = .025, φ = .132) and between Groups 3 and 4 (*p* = .025, φ = .240).

### Social isolation and depression trajectories predicting cognitive impairment at age 59

**Figure 2.**
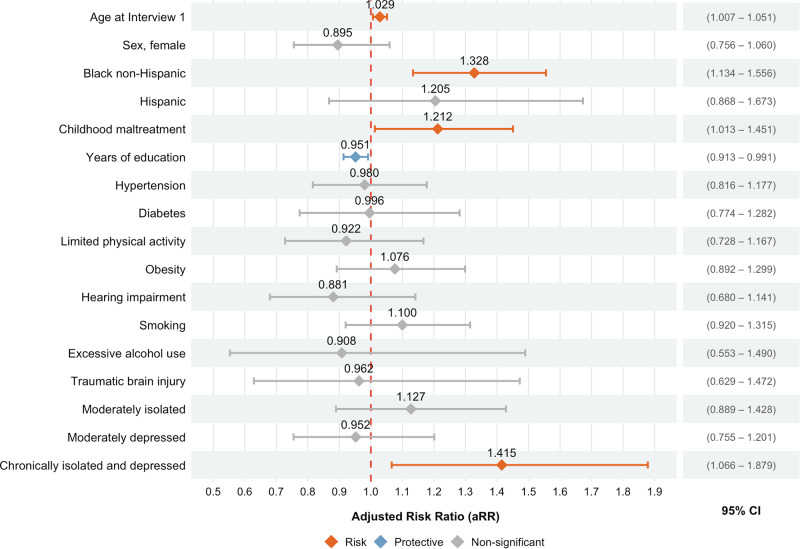
Adjusted risk ratios of CIND at age 59 by childhood maltreatment and social isolation/depression trajectory groups. Figure 2 long description.

*Note.* Estimates are from modified Poisson regression models. GBTM reference category, Group 1 = Not isolated or depressed. Sex: male (ref.). Race/ethnicity, White non-Hispanic (ref.)

As depicted in Figure 2, the results from the modified Poisson regression models identified age at Interview 1, Black non-Hispanic, years of education, childhood maltreatment and membership in the Group 4 (‘Chronically isolated and depressed’) trajectory of social isolation and depression as significant predictors of future CIND at age 59. Specifically, greater age, being Black non-Hispanic and having experienced childhood maltreatment increased the risk of CIND, whereas higher levels of education were protective. Membership in Group 4 (‘Chronically isolated and depressed’) was also associated with an increased risk of CIND. No other significant predictor was identified.

The adjusted risk ratio (aRR) for age at Interview 1 was 1.029 (95% CI [1.007, 1.051]), indicating that each additional year of age is associated with a 2.9% increase in the risk of CIND. The aRR for the Black non-Hispanic group was 1.328 (95% CI [1.134, 1.556]), indicating that compared to White non-Hispanic participants, Black non-Hispanics in this cohort have a 32.8% increase in the risk of CIND. The aRR for childhood maltreatment was 1.212 (95% CI [1.013, 1.451]), indicating that individuals who experienced childhood maltreatment have a 21.2% higher risk of CIND compared to those who did not have documented histories of childhood maltreatment. The aRR for years of education was 0.951 (95% CI [0.913, 0.991]), suggesting that each additional year of education is associated with a 4.9% reduction in the risk of CIND. Membership in Group 4 compared to Group 1 was associated with a significantly higher risk of CIND, with an aRR of 1.415 (95% CI [1.066, 1.879]), suggesting that individuals in Group 4 have a 41.5% increased risk of CIND relative to those in Group 1. All other predictors were non-significant. The delta-adjusted sensitivity analysis yielded broadly similar results. The association between trajectory Group 4 and CIND attenuated as the assumed departure from MAR increased, but it remained statistically significant across all scenarios examined, suggesting that the finding is reasonably robust to MNAR departures, albeit its magnitude is sensitive to the strength of the assumption.

### Snapshots of social isolation and depression predicting cognitive impairment at age 59

**Figure 3.**
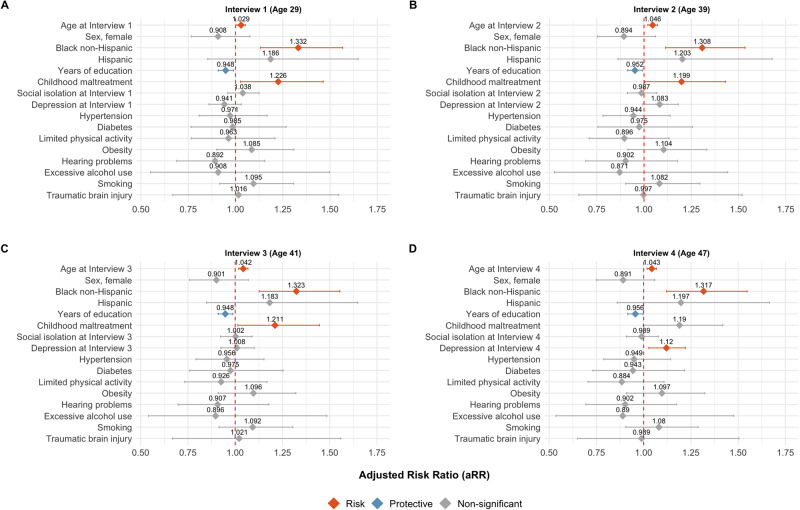
Adjusted risk ratios for CIND at age 59 by childhood maltreatment, social isolation, and depression at each interview. Figure 3 long description.

*Note*. Estimates are from modified Poisson regression models. Sex: male (ref.). Race/ethnicity, White non-Hispanic (ref.)

Figure 3 illustrates the aRRs and CIs for each predictor. Age at each interview was significant in all four interviews, as were years of education. These effects were relatively stable across interviews: each additional year of age increased the risk of CIND by approximately 3–5%, and each additional year of education decreased the risk by approximately 4–5%. Social isolation was not a significant predictor in any of the four interviews. Depression was not significant at Interviews 1 through 3, but became significant at Interview 4, increasing the risk of CIND by 11.2% (aRR = 1.120, 95% CI [1.028, 1.221]). Black non-Hispanics (relative to White non-Hispanics) had a significantly increased risk of CIND across all four interviews, with consistent aRRs between 1.310 and 1.340. Group 3 membership was significantly associated with increased CIND risk at Interviews 1 through 3 (aRR ranged from 1.199 to 1.225), but not at Interview 4. All the other variables were not significant predictors at any interview.

## Discussion

Overall, our findings supported both hypotheses. Documented childhood maltreatment was associated with an increased risk of CIND at age 59 (H1), and membership in the highest-risk trajectory (Group 4, ‘chronically isolated and depressed’) was associated with an increased risk of CIND at age 59 compared with membership in the no-risk trajectory (H2).

Highlighting the cumulative effects of social isolation and depression, Group 4 (‘Chronically isolated and depressed’) stands out with the highest prevalence of CIND and is the only trajectory group significantly linked to increased CIND risk at age 59. In contrast, Groups 2 (‘Moderately isolated’) and 3 (‘Moderately depressed’), despite experiencing low to moderate levels of isolation and depression, did not show an increased risk of CIND compared to Group 1 (‘Not isolated or depressed’). This suggests a degree of resilience among individuals in Groups 2 (‘Moderately isolated’) and 3 (‘Moderately depressed’). The persistence of group differences after adjustment for other risk factors for neurodegenerative outcomes suggests that the chronic co-occurrence of social isolation and depression is associated with cognitive risk beyond that accounted for by the measured characteristics.

Prior research indicates that adults with histories of childhood maltreatment are more likely to experience social isolation and depression (Widom *et al.*, 2007; Gilbert *et al.*, 2009; Gardner *et al.*, 2019; Maxfield *et al.*, 2023), both of which have been associated with an elevated risk of cognitive impairment and dementia (O’Brien, 2004; Rock *et al.*, 2014; Kuiper *et al.*, 2016; Evans *et al.*, 2019; Piolatto *et al.*, 2022; Laustsen *et al.*, 2024; Veronese *et al.*, 2024). Findings from this cohort suggest that childhood abuse and neglect may have long-standing and interrelated social, emotional and cognitive consequences. It has been hypothesized that childhood maltreatment negatively affects brain development (Teicher *et al.*, 2016) and may have downstream effects on cognitive development and academic performance (Watts‐English *et al.*, 2006). In the present study, childhood maltreatment and chronic co-occurrence of social isolation and depression were each independently associated with CIND in late midlife after adjustment for other relevant risk factors. The precise timeline of each consequence of maltreatment is complicated by how these social, emotional and cognitive outcomes may reinforce one another. Nevertheless, recognizing the potentially additive nature of risk factors for CIND is an important step toward early identification of, and intervention for, individuals at greatest risk. Further research should address what factors distinguish individuals experiencing moderate versus chronic social isolation and depression. Early-life contributors to cognitive reserve resilience remain underexplored and warrant further inquiry, particularly regarding cognitive reserve formation and retention through late-life (Clouston *et al.*, 2020).

Sex differences in cognitive impairment literature are inconsistent across cohort studies; in this cohort, sex was not associated with a higher risk of CIND. Some longitudinal research has found that, compared with women, men often perform lower at baseline on several cognitive domains and may experience faster declines, such as steeper deterioration in global cognition, episodic memory and language fluency (Morley, 2018). However, other cohorts have reported that women experience faster declines in global cognition and executive function than men, whereas men may also show steeper verbal memory decline in normal ageing (Levine *et al.*, 2021). This heterogeneity can depend on many factors, such as baseline cognitive reserve, age structure, comorbidities, sex hormones, cardiovascular diseases, genetics, lifestyle and psychological factors (Mielke *et al.*, 2014; Baumgart *et al.*, 2015). Thus, these divergent findings likely reflect variation in study populations, assessment tools and the specific cognitive domains examined.

Another key finding in the literature is that MCI is delayed and compressed toward the end of life for advantaged groups, particularly White individuals and those with higher levels of education (Hale *et al.*, 2020; Bai *et al.*, 2022). This aligns with the findings of this study, as White non-Hispanic participants and those with more years of education exhibited a substantially lower risk of CIND at age 59. Conversely, previous epidemiological research in the US has found that disadvantaged groups, such as Black or African-American individuals and those with fewer years of education, experience an earlier onset, higher lifetime risk and a longer duration of MCI (Mayeda *et al.*, 2016; Farina *et al.*, 2020). This disparity may be explained by multiple factors reflecting the impact of structural racism and educational inequities, including socioeconomic deprivation, neighbourhood disinvestment and disparities in healthcare access and treatment-seeking behaviour (Viner *et al.*, 2012; Hale *et al.*, 2020; Kornblith *et al.*, 2022). Further investigation is needed to examine the underlying causes of these disparities among disadvantaged groups and how disparities may be exacerbated by childhood maltreatment (Currie and Widom, 2010; Pinto Pereira *et al.*, 2017). Potential contributing factors include the role of discrimination in cognitive reserve (Stern, 2009), occupational hazards, gender disparities, educational opportunities and other social determinants of health, as well as medical decision-making biases that may exacerbate these differences (Viner *et al.*, 2012; Kornblith *et al.*, 2022).

## Limitations

Several limitations of this study should be acknowledged. The cases analysed were identified through the courts, restricting the generalizability of the findings to instances of maltreatment that go unreported or are not substantiated by authorities. The predominantly low socioeconomic status of the sample further limits the applicability of these results to abuse and neglect occurring in middle- and upper-class families. The study reflects the experiences of children who grew up in the Midwest during the late 1960s and early 1970s, and it may raise concerns about the relevance of these findings to contemporary society. Nonetheless, the maltreatment cases included in this research are similar to those currently handled by the child protection system and judicial authorities. Depressive symptoms were not measured with the same instrument at all four time points. The baseline interview used the NIMH DIS-III-R, and then considered the gold-standard structured psychiatric interview for large community samples, to establish baseline mental health and social, behavioural, and cognitive functioning. Later interviews captured depression through the CES-D, so we standardized depression scores at each wave to allow comparability. The social isolation measure showed modest internal consistency, which is not uncommon for short scales, and the binary recoding of the individual items due to the skewed response distribution may have reduced sensitivity in capturing variation. We also acknowledge that depression and social isolation may share some conceptual overlap, particularly regarding symptoms related to social withdrawal. The research-based classification of CIND was selected because the available data did not include the information needed to rule out medical causes, as an MCI diagnosis requires. MCI is a multifactorial and complex condition characterized by cognitive impairment in the absence of functional decline, in contrast to dementia, which is defined by the presence of functional impairment. A key challenge in diagnosing MCI is the absence of universally applied criteria, with varying definitions that must balance the risks of false negatives and false positives (Petersen, 2004). However, it is important to identify CIND, given its association with a higher risk for dementia (Tuokko *et al.*, 2003; Chertkow *et al.*, 2007). Finally, although the models adjusted for most established risk factors, the apolipoprotein E (APOE) genotype was not available in this cohort and could not be included. Future work should continue to examine the tested associations with more comprehensive measures.

## Conclusion

This study suggest that childhood maltreatment and chronic trajectories of moderate to high social isolation and depression contributed to higher rates of CIND in midlife. Black non-Hispanics had a substantially increased prospective risk of CIND. Each additional year of education significantly decreased risk, consistent with evidence that the level of education and continuing learning activities build cognitive reserve and help protect against early decline (Matyas *et al.*, 2019).

These findings highlight the importance of prospective longitudinal assessments and indicate that individuals experiencing chronic isolation and depression in young adulthood and midlife may represent a critical target for early intervention. Promoting social engagement, physical activity, cognitive training and adequate sleep may help mitigate risk (Gorelick, 2018). Future research should also integrate heritability, biomarkers and lifestyle factors to clarify the mechanisms linking childhood adversity to later emotional and behavioural outcomes that contribute to CIND (Kivipelto *et al.*, 2018). Supporting individuals at heightened risk in late midlife may help preserve cognitive health and potentially delay progression to more severe cognitive impairment.

## Data Availability

The data reported in the current article are not publicly available because they contain extremely sensitive information that could compromise research participant privacy and confidentiality. We cannot provide individual-level data from this project because our confidentiality agreement with the participants in this study precludes this. The data are available on request from qualified scientists. Requests require a concept paper describing the purpose of data access, ethical approval at the applicant’s university, and provision for secure data access.

## Table 1 Long description

The table summarizes participant characteristics from court records and five interview waves through 2022–2023. Sample size declined from 1,575 in the records to 447 at Interview 5, while mean age increased from 29.2 to 59.4 years. Social isolation fluctuated modestly, whereas depression symptoms were higher in later interviews and peaked at Interview 5. At Interview 5, 266 participants, or about 63%, had mild cognitive impairment. The sample remained predominantly White non-Hispanic and Black non-Hispanic, with modest shifts in sex and race/ethnicity composition over time.

Navigate back to Table 1.

## Table 2 Long description

The table compares model-fit and classification quality across two- through seven-group solutions using AIC, BIC, SSABIC, two likelihood-ratio test p-values, entropy, smallest group size, and average posterior probability ranges. AIC decreases steadily as more groups are added . Similarly, BIC is lowest for the 7-group solution. SSABIC is lowest for the 5-group solution, then increases for the 6-group and 7-group solution. The adjusted Adj. LMR-LRT p is significant for two, four and five groups (.0073 and .0097) but not for three, six, or seven groups, while the bootstrap likelihood-ratio test is below .001 for all solutions. Entropy is highest for the 2-group solution at .781 and ranges from .72 to .77 for three to seven groups. The smallest group proportion drops from 16.30% with two groups to 1.92% with seven groups, with higher-group solutions creating very small classes. Average posterior probability ranges are generally high across the different solutions .

Navigate back to Table 2.

## Table 3 Long description

The table compares participant demographics, childhood maltreatment history, and adult health conditions across the four trajectory groups of social isiolation and depression. Group 1 is the largest (810 participants), while Group 4 is the smallest (49 participants). Average interview age is about 60 years in all groups, but Group 3 and Group 4 include more women (64.0%and 59.2%) than Groups 1 and 2 (about 45% ). Education is highest in Group 1 (mea 11.8 years) and lowest in Group 4 (mean 10.1 years). Childhood maltreatment is most common in Group 2 (73.2%) and remains high in Groups 3 and 4 (67.2%and 64.6%). Several health risks are especially higher in Group 4. All these comparisons are exploratory.

Navigate back to Table 3.

## Figure 1 Long description

Group 1 (Not isolated or depressed, ~68%): slightly below average on both isolation and depression, essentially flat, no risk group. Group 2 (Moderately isolated, ~12%): elevated isolation but low or no depression, with isolation gradually declining over time. Group 3 (Moderately depressed, ~16%): low isolation with moderately elevated depression that rises early then plateaus. Group 4 (Chronically isolated and depressed, ~4%): high on both, peaking in the late 30s to early 40s before easing slightly by age 49.

Navigate back to Figure 1.

## Figure 2 Long description

CIND at age 59 was significantly associated with older age at Interview 1, Black non-Hispanic race/ethnicity, childhood maltreatment, and membership in the chronically isolated and depressed trajectory group. More years of education was protective.

Navigate back to Figure 2.

## Figure 3 Long description

For the four-panel forest plot illustrating the snapshots from each interview, the findings were mostly consistent across time. At Interviews 1, 2, and 3, older age, Black non-Hispanic race/ethnicity, and childhood maltreatment were associated with higher risk of later CIND, while more years of education was protective. Social isolation and depression at those interviews were not significantly associated with later CIND. At interview 4, older age, Black non-Hispanic race/ethnicity and depression emerged as a significant predictors.

Navigate back to Figure 3.
